# 3D Polyaniline Architecture by Concurrent Inorganic and Organic Acid Doping for Superior and Robust High Rate Supercapacitor Performance

**DOI:** 10.1038/srep21002

**Published:** 2016-02-12

**Authors:** Yogesh Gawli, Abhik Banerjee, Dipti Dhakras, Meenal Deo, Dinesh Bulani, Prakash Wadgaonkar, Manjusha Shelke, Satishchandra Ogale

**Affiliations:** 1 Physical and Materials Chemistry Division, National Chemical Laboratory (CSIR-NCL), Pashan, Pune, 411008, India; 2 Academy of Scientific and Industrial Research, (AcSIR) Anusandhan Bhavan,2, Rafi Marg, New Delhi, India; 3 Department of Physics and Centre for Energy Science, Indian Institute of Science Education and Research (IISER), Dr. Homi Bhabha Road, Pune 411008, India

## Abstract

A good high rate supercapacitor performance requires a fine control of morphological (surface area and pore size distribution) and electrical properties of the electrode materials. Polyaniline (PANI) is an interesting material in supercapacitor context because it stores energy Faradaically. However in conventional inorganic (e.g. HCl) acid doping, the conductivity is high but the morphological features are undesirable. On the other hand, in weak organic acid (e.g. phytic acid) doping, interesting and desirable 3D connected morphological features are attained but the conductivity is poorer. Here the synergy of the positive quality factors of these two acid doping approaches is realized by concurrent and optimized strong-inorganic (HCl) and weak-organic (phytic) acid doping, resulting in a molecular composite material that renders impressive and robust supercapacitor performance. Thus, a nearly constant high specific capacitance of 350 F g^−1^ is realized for the optimised case of binary doping over the entire range of 1 A g^−1^ to 40 A g^−1^ with stability of 500 cycles at 40 A g^−1^. Frequency dependant conductivity measurements show that the optimized co-doped case is more metallic than separately doped materials. This transport property emanates from the unique 3D single molecular character of such system.

In light of the growing energy storage requirements of the electrically driven modern world, supercapacitors represent one of the most intensely researched topic in recent years because of their high power delivery capability in addition to a fairly high energy density^1^. Supercapacitors cover an interesting and important application space where the slow charging/discharging weakness of battery systems is an undesirable limitation. A number of material systems are being designed and synthesized which could serve well as electrode materials in supercapacitors^2^. These are needed to be highly porous with hierarchical pore size distribution and high electrical conductivity. Carbon based materials including graphene, CNT *etc*. mainly store charges on their surface by forming an electric double layer (EDLC)^3^,^4^,^5^,^6^,^7^,^8^, unless presence of dopants add a Faradaic reaction component to the mechanism. Many oxides, sulphides and conducting polymeric systems can also store charge in the surface layers through Faradaic reactions (pseudocapacitor), which offer a benefit of de-solvation of the adsorbing/reacting ions and therefore higher energy density of energy storage than the EDLC devices^9^,^10^. However the requirements of hierarchical porosity and high conductivity remain the same as for the EDLC case.

Pseudocapacitors are made of metal hydroxides such as Ni(OH)_2_^11^, oxides like NiO^12^, MnO_2_^13^, RuO_2_^14^, metal sulphides like NiCo_2_S_4_^15^, metal selenides like Co_0.85_Se^16^ or conducting polymers such as PANI^17^,^18^,^19^,^20^, polypyrrole^21^,^22^, *etc*. Metal oxides and sulphides possess high theoretical capacitance but they lack adequate conductivity for charge transportation towards the current collector and most of the corresponding metals such as nickel and ruthenium are expensive^23^. Moreover it is non-trivial to fabricate flexible devices using these inorganic systems. On the other hand conducting polymers (CPs) like PANI not only possess high theoretical capacitance^24^ but can also be easily processed to make flexible, roll-able devices^15^,^16^,^17^,^18^,^19^,^20^,^21^,^22^,^23^,^24^,^25^,^26^,^27^,^28^. Furthermore, the corresponding precursors are cheaper than metals.

In the case of polymeric systems which is the topic of this work, morphologies are often difficult to control in the desired forms and they also influence the transport properties in several ways^29^,^30^. The coiling of polymers or agglomeration of polymer chains^31^ for instance, can hamper transport by influencing both the intra-chain hopping and inter-chain tunnelling. Even if uniform homogeneously intermingled oriented chain morphology is attained the inter-chain transport is still mediated by sluggish tunnelling processes. If one were to develop a scheme to make a molecularly interconnected 3D network of conducting polymeric chains with the desired multilevel porosity, one could envision a material capable of rendering a high rate robust super-capacitive performance^32^. This indeed has been the motivation for the present work and the realization of the stated goal is amply demonstrated through careful optimization experiments on a scheme of binary (strong-inorganic + weak-organic) acid doping in the conducting polymer polyaniline. There are two previous reports by Kuo *et al.*^33^,^34^ on dual acid doping in polyaniline but in a different context, namely methanol oxidation with Pt nanoparticles loaded PANI as a catalyst material. They co-doped polyacrylic acid (PAA) and HCl which was shown to lead to effective dispersion of Pt nanoparticles in such polymer matrix as against only HCl doped PANI due to better morphology. In our case the morphology as well as electrical conductivity are very important factors and we show that both can be optimized and enhanced via controlled binary doping of phytic acid and HCl.

PANI is the most studied conducting polymer amongst its class because of its environmental stability, rich redox chemistry *etc*^35^,^36^,^37^,^38^. Since it shows potential dependent variable oxidation states, interconversion of these leads to a very high theoretical capacitance value. Literature shows that ample amount of research has been performed using PANI in the supercapacitor application^39^ which depends on morphology^40^,^41^. Its morphology as well as the electrical and physical properties depend on synthesis conditions^42^,^43^,^44^ especially on the type of acid dopant used. For instance, the HCl doped PANI, (low pH < 2) is more conducting as compared to the cases of other dopants because it is a strong acid (Chlorine is highly electronegative) and hence doping (delocalization of electrons) is effective as compared to doping with weak organic acids like acetic acid, phytic acid (PA) (here, oxygen is lesser electronegative than Cl) *etc*. Certain carboxylic acids (high pH ~3–6) like benzene tricarboxylic acid when used as dopants provide nanotubes of various dimensions^45^, whereas the phosphoric acid derivatives such as PA being a multidentade dopant leads to a 3D interconnected nanofibrous structure.

In spite of several efforts during the past decades, PANI suffers from two major problems in the context of charge storage applications: First is the charge storage stability *i.e.* its capacitance at a particular current density dies out rapidly over thousand cycles or so. The second problem is that its capacitance fades away as the charge/discharge current density is increased. It has very high capacity at 1 A g^−1^ but it decreases considerably as the current density is increased to 10 A g^−1^ and above. Different solutions to the first problem have been suggested by Liu *et al.* and Vonlanthen *et al.* Liu *et al.*^46^ coated the PANI electrodes with a very thin (3 nm) layer of carbon hydrothermally, and the capacitance value was shown to remain stable for 10k cycles. Vonlanthen *et al.*^47^ introduced a second redox system to tune the electron transfer processes at the PANI electrode which eliminated unfavourable processes occurring at the PANI electrode and showed stability up to 50k cycles. Solving the second problem and coupling it with the solution(s) of the first problem could bring PANI one big step closer to being state of the art material in the context of charge storage.

In the present work we provide a prototype solution to the second problem via co-doping. Thus, we simultaneously dope PANI with two different (one weak-organic and another strong-inorganic) protonic acid dopants, namely phytic acid (PA) and hydrochloric acid. These two dopants furnish a complementary set of electrochemical and morphological properties to PANI chains. The PA renders inter-chain doping^48^ whereas HCl renders intra-chain doping. PA gives a 3D interconnected nanofiber structure to the PANI backbone along with an easy access to the ionic species via a well-formed and distributed pore structure, but a lower electrical conductivity because of its weak acid character. HCl on the other hand, when doped alone, is known to yield much higher conductivity. We simultaneously dope these two dopants on polymer chains resulting into binary doped polymer as shown in Fig. 1. Due to the existence of 3D structure all the PANI chains can be accessed at once. Moreover our synthesis procedure is template free, does not require high-end engines to synthesize, scalable and less expensive, hence commercially practical.

The stereochemistry of dopant is one of the important factors which determines the morphology of the resulting polymer since the steric nature of dopant and its locality can determine stress, relaxation and distance between nearby polymer chains^49^. It can also determine the extended or coiling tendency of the polymer chains. Phytic acid anion is an organic bulky group having the ability to interact with several chains at a time, hence it is expected that its tendency towards forming coiled structure is lower than small and non cross-linking anions such as chlorides, sulphates and nitrates.

## Results and Discussion

It is a well-established fact that polymerisation of aniline is facile in high dielectric polar media such as water because the product is not soluble and also water promotes nanofibrilar growth at nuclei level^50^. On the other hand phytic acid, the cross-linker, has very high affinity for water and can easily dissolve aniline in its solution at room temperature. Phytic acid is insoluble in organic solvents such as dimethyl formamide, N-methyl-2-pyrrolidone and ethanol, hence water was chosen for our co-doping synthesis.

**Figure 2:**
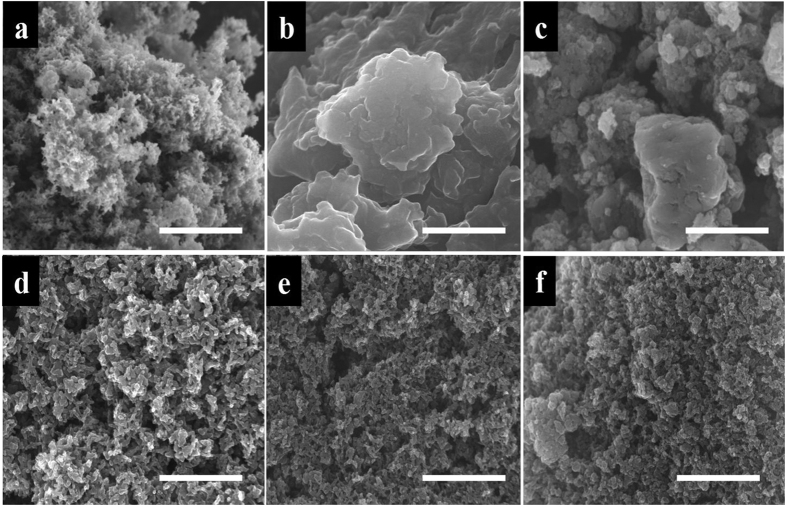
Scaning electron microscope images of all materials, Scale bar 5 μm: (**a**) PANI PA (**b)** PANI HCl **(c**) PANI 20PA (**d**) PANI 45PA (**e**) PANI 60PA (**f**) PANI 80PA.

As stated earlier, the strong-inorganic acid doping renders high electrical conductivity but undesirable morphology, while the weak-organic acid doping leads to a desirable morphology but lower electrical conductivity. Hence binary acid doping was performed in different proportions of the inorganic and organic acid components. Figure 2 shows the different morphologies attained for different acid proportions. An evolution from dense nanoscale morphology for PANI HCl to a 3D connected open-type porous morphology for PANI PA is clearly noted. In the case of PANI HCl a nano-structure assembled lump like morphology is seen while for interconnected fibrous morphology with several entangled fibers with some bigger ones. The desirable morphology-type with hierarchical pore size distribution was noted to evolve just around PANI 45PA.

**Figure 3:**
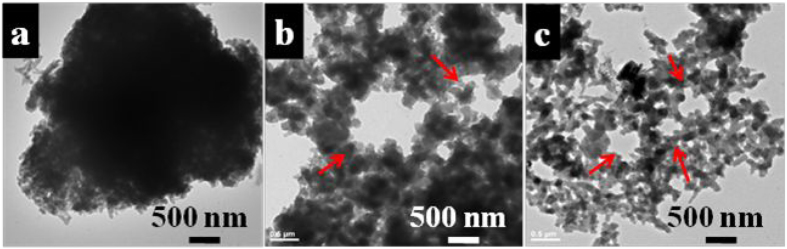
Transmission electron microscope images of (**a**) PANI HCl, (**b**) PANI PA, (**c**) PANI 45PA samples.

The TEM images for three specific cases of interest are shown in Fig. 3. PANI HCl (Fig. 3a) case confirms the nanoassembled lumpy morphology that is revealed by FESEM. Figure 3b corresponding to the PANI PA sample exhibits extended structures with ill-defined particle feature size distribution and connectivity. Few micron scale big pores are seen with some smaller ones. Interestingly, in the case of PANI 45PA (Fig. 3c) it is noted that some morphological features (e.g. several hundred nm scale pores, marked with red arrows) are similar to those of PANI PA, but the nanoscale constitution is far more uniform and rod-like as compared to that in PANI PA. This gives PANI 45PA an interesting hierarchical pore structure that is absent in the case of both the other forms namely PANI HCl and PANI PA. Such branched interconnected fibre-type morphology with hierarchical pore structure is known to be of great value to the charge storage application such as a supercapacitor, and our data illucidates this clearly as discussed later in the text. Additional TEM images for PANI 45PA are given in supplementary as Fig. S5. Figure S1 shows the SEM image of PANI 45PA alongside its elemental mapping of the two elements phosphorous (belonging to PA) and chlorine (from HCl). The mapped image shows homogeneous distribution of both the elements indicating uniform dispersion of dopants throughout the chains. Figure S2 presents the FTIR data for the two polymers PANI PA, PANI 45PA. The building units of polyaniline i.e. quinonoid and benzenoid show similar C-C stretching frequency viz. 1483 cm^−1^ and 1561 cm^−1^ for the two materials. Also, the absorption at 1242 cm^−1^ which represents the C-N^+.^ frequency mode of the polaronic lattice^51^ (a result of protonic acid doping) is also as expected. Hence it can be rationalized that PANI 45PA also has the emeraldine state like PANI PA.

**Figure 4:**
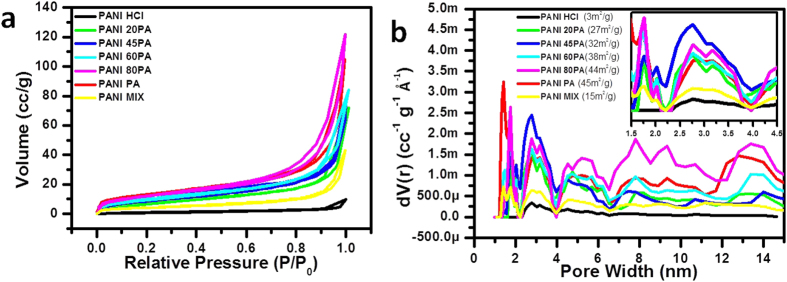
**(a)** Adsorption Isotherms for the four materials of interest, **(b**) pore size distribution (inset zoom at lower pore width).

Figure 4a shows the nitrogen adsorption isotherms for all the samples. Also shown is the data for the case of just a physical mixture of PANI HCl and PANI PA in the same proportion 55:45 for comparison purposes. PANI PA has the highest surface area of 45 m^2^ g^−1^, while PANI HCl has the lowest (3 m^2^ g^−1^). Since PANI MIX is the physical mixture of these two, its isotherm lies midway between its two parent materials. It is to be noticed that surface area increases as the PA concentration increases. Interestingly, all the binary doped samples have significantly higher surface areas than PANI HCl, and are even higher than PANI MIX. This reflects positively upon the 3D molecular architecture with hierarchical porosity resulting from the special synthesis of binary doped samples over a simple physical mixture. The pore size distribution for all the samples is plotted in Fig. 4b. The trend in the case of pore volume is similar to the evolution of the nitrogen adsorption isotherm. PANI PA has the highest pore volume consistent with the surface area. The interesting point to note is that PANI HCl does not show any reasonable porosity (pore distribution) in clear contrast to PANI PA and has the smallest pore volume. Most importantly PANI 45PA (which has almost half of the HCl doped PANI chains, which enhance conductivity) has even a higher pore density in the range of 2–4 nm than PANI PA and comparable in the range of 4–6 nm.

Due to crosslinking nature of PA, polymerisation of aniline leads to interconnected fiber structure. During polymerisation if few of the PA molecules are replaced by much smaller molecule like HCl, typically in the cases of PANI 80PA and PANI 45PA pore volume should relatively increase which can be spotted in Fig. 4b in the range of 2–4 nm, but further such replacement leads to decrease the extent of crosslinking and results in either comparable or lower pore volume which is observed for PANI 60PA and PANI 20PA. This confirms that because of its high pore volume in that range, PANI 45PA is structurally favourable for charge storage over other samples.

Tapping density (TD) is the measure of fluffiness of the material. Higher the TD more the compactness. Figure S3 shows the TD for all the materials as a function of % PA. PANI HCl has the highest TD while PANI PA has the least TD. This can be related to the surface area of these materials. Generally, higher the surface area higher is the fluffiness and less is the density. PANI 45PA has the intermediate value and those with higher PA have lower values. In our context, binary doping drops the density which is necessary for supercapacitor application.

**Figure 5:**
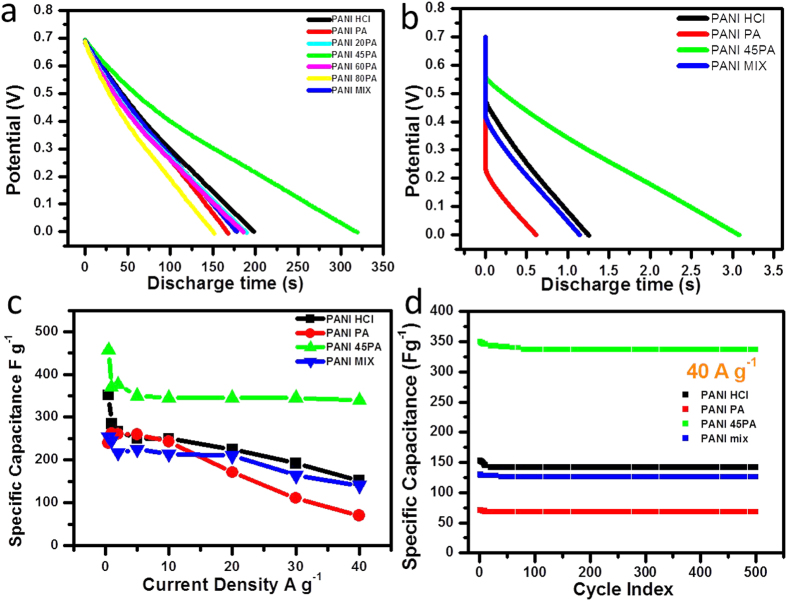
**(a)** Discharge curves for all the samples of interest at 5 A g^−1^, (**b)** Discharge curves of the four samples at 40 A g^−1^, **(c)** Specific capacitance of the four samples at selected current densities, **(d)** Cycling stability of 4 selected samples at 40 A g^−1^.

Figure 5a displays the discharge time curves for all samples at lower current density of 0.5 A g^−1^ in two electrode system. This includes PANI HCl, PANI PA, PANI MIX and PANI xPA (x = 20, 45, 60, 80, 100). The specific capacitance was calculated from discharge time by the following equation 1 having symbols of usual meaning:


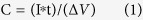


It is observed that the PANI 45PA exhibits higher capacitance (457 F g^−1^) as compared to PANI HCl (352 F g^−1^), PANI PA (240 F g^−1^), their physical mixture (254 F g^−1^) as well as all binary doped PANI. Based on this and the pore size distribution data, in the following sections only on this optimised case is focussed for further comparison(s).

The PANI 45PA sample and the PANI HCl, PANI PA and PANI MIX samples were all subjected to discharge study at much higher current density of 40 A g^−1^. The discharge time (t) plots are shown in Fig. 5b. The order found is as follows: PANI PA < PANI MIX < PANI HCl < PANI 45PA. It can be seen that the general trend observed for 0.5 A g^−1^ is maintained here also. PANI 45PA shows the highest specific capacitance of ~350 F g^−1^ as compared to both PANI HCl and PANI PA which have the values as 250 F g^−1^ and 260 F g^−1^, respectively.

These selected four materials were tested at various discharging current densities ranging from 0.5 A g^−1^ to 40 A g^−1^. From their discharge time curves the respective specific capacitance values of four selected samples were calculated and plotted in Fig. 5c. For PANI HCl the capacitance value is 352 F g^−1^ at 0.5 A g^−1^ which matches with the literature, and it gradually decreases to 152 F g^−1^ at 40 A g^−1^ indicating a total loss of 200 F g^−1^. The loss is less (140 F g^−1^) for the case of PANI PA and is the least (~110 F g^−1^) for the optimised PANI 45PA case. Another important point to appreciate is that in the case of PANI 45PA the specific capacitance value (~350 F g^−1^) varies only very little over a whole broad range of current densities i.e. 1 A g^−1^ to 40 A g^−1^. PANI PA and PANI MIX show a much poorer result amongst all the materials. Thus PANI 45PA is characteristically unique as compared to the physical mixture. The individual discharge time for all the current densities for PANI HCl, PANI PA, PANI 45PA and PANI MIX are shown in Fig. S4. In the range of 1 A g^−1^ to 40 A g^−1^ almost 90% of capacitance retention takes place in the case of PANI 45PA whereas in the case of PANI HCl and PANI PA the retention is only 53% and 26%, respectively. This high rate performance is certainly superior than various other reports for PANI based supercapacitors, which include morphology dependent electrochemical behaviour and samples involving composites with graphene. The detailed comparison is given in Table S1.

Further, the selected four samples were subjected to charge-discharge for 500 cycles at a high current density of 40 A g^−1^. Figure 5d shows a significant stability for all the samples without loss of any appreciable amount of capacitance even at such high rate. To the best of our knowledge, such ~99% stability of 350 F g^−1^ of PANI 45PA has never been demonstrated in prior reports on PANI, specifically in two electrode system which is more practical approach than the three electrode case.

**Figure 6:**
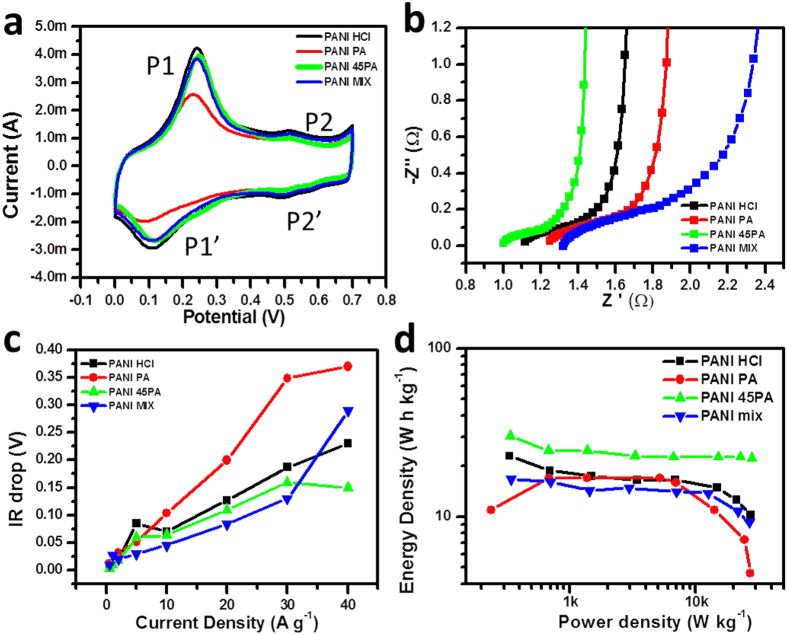
**(a)** Cyclic voltammetry of selected four samples depicting redox process, **(b)** Electrochemical Impedance Spectroscopy (EIS) plots for the selected four materials, **(c)** IR drop variation as a function of current density, **(d)** Ragone plot.

Cyclic voltammetry was done in three electrode system to understand the underlying processes. The cyclic voltammogram of PANI HCl, PANI PA, PANI 45PA and PANI MIX measured at 5 mV s^−1^ is shown in Fig. 6a. They all have a typical emeraldine salt nature^32^. All these samples had the same loading. The transition redox peaks of the four samples almost coincide with a little shift especially in the case of PANI PA. The first redox pair P1-P'1 corresponds to the leucoemeraldine to emeraldine and the other P2-P'2 corresponds to the emeraldine to pernigraniline phase. The peak-to-peak separation for a pair of redox peaks indicates the reversibility of the material for this measurement. For the pair P1-P'1, the peak-to-peak separation for PANI PA is the highest of 0.18 V, whereas it is 0.14 V for PANI HCl and importantly the least, 0.12 V, for the optimised PANI 45PA case. This confirms the higher reversibility for the optimised case. The CV nature of PANI MIX resembles that of PANI HCl having current values in between those for the parent materials which are different from PANI 45 PA.

Figure 6b shows the Nyquist plots for the four samples as said above. The fitted circuit is shown in Fig. S5. The electrical circuit consists of 3 important elements. The first is the charge transfer resistance R_ct_ which is the diameter of the semicircle at low frequency region^51^. It can be realised that PANI MIX has highest R_ct_ of ~1.300 Ω, whereas PANI 45PA has the least R_ct_ of ~420 mΩ value. This implies deeper diffusion of ions in the interior of the optimized material as compared to the other cases. The second important parameter is the series resistance (SR) which is obtained from the intersection of this semicircle on the X axis. This SR designates the resistance corresponding to the interface of the electrode-electrolyte and the contact resistances. Lesser SR indicates better access for the ions towards the electrode. The trend, once again favours PANI 45PA over other cases, with the least value of 0.900 Ω as seen from Table 1. It also shows the values of the individual knee frequency, the third key parameter^52^. This value separates resistive and capacitive behaviour on the plot. Higher value implies better supercapacitor performance of such material, since below this frequency the capacitive nature dominates. PANI 45PA has the highest value of ~16 Hz, which is not only better than the previously reported values, but much superior than the values for PANI HCl, PANI PA and PANI MIX namely 7 Hz, 4 Hz and 2.9 Hz respectively. Four probe method was also used to measure the true DC conductivity values. PANI HCl, PANI PA, PANI 45PA and PANI MIX showed the conductivity values of 0.48, 0.32, 0.61 and 0.43 S cm^−1^, respectively. This clearly points to the overall superior performance of PANI 45PA.

To better understand the events taking place at higher discharging current densities the effect of various discharge current densities on the IR potential drop was studied. The internal resistance of the assembly plays critical role in the potential drop and in turn the energy and power density^53^. Higher is the potential drop lesser is the potential window available for use and lesser is charge storage. Hence it is desirable to have the least potential drop. Figure 6c depicts the plot of potential drop at each discharging density for the four samples of interest. The optimized case has the least potential drop at the highest current density, which implies that more Faradaic centres are available to the electrolyte in this case as compared to the other cases. The IR drop data correlates quite well to morphological features. Due to the presence of both type of pores in the case of PANI 45PA, specially the higher density of nanoscale pores, the accessibility for the ions is highest which leads to minimum loss of useful voltage, hence the highest specific capacitance. It is worth mentioning that the potential drop value for PANI 45PA case (~0.15 V) is almost half that of PANI PA (~0.36 V) at 40 A g^−1^. These data along with EIS measurement confirms that PANI 45PA has the easiest accessibility for ions even at a current density as high as 40 A g^−1^. One additional point to consider is the specific nature of the PANI MIX data. Initially the IR drop is lower may be due to the presence of PANI HCl groups but as current density increases to the highest value, due to the hostile co-ordination between PANI HCl and PANI PA, the IR drop dramatically intensifies which is even higher than that of PANI 45PA. This again supports the fact that PANI 45PA is not the physical mixture like PANI MIX and it does involve simultaneous doping.

Ragone plot is the best way to represent the energy density and power density, based on which all energy storage systems can be differentiated and assessed. These values were calculated from the specific capacitance^23^. Figure 6d shows the plots for the four samples of interest. It can be realized that PANI HCl has more energy density than PANI PA at all current densities; on the other hand PANI PA has comparable and higher power density at low current densities. These quality factors are partially imitated by PANI 45PA resulting into a synchronised enhancement of power and energy density to 28 kW kg^−1^ and 22.4 Wh kg^−1^ respectively. PANI MIX does not show such synchronisation and shows only intermediate values. Additional point to be noted from the Ragone plot is that except PANI 45PA, all the other samples loose the energy density as the power density is increased. Amongst all these, PANI PA shows the largest decrease due to insufficient conductivity required to respond at high power. PANI 45PA on the other hand shows an almost negligible decrease in energy density for a broad range of power densities which is the most important characteristic of this material and not observed previously for any polyaniline based systems.

**Figure 7:**
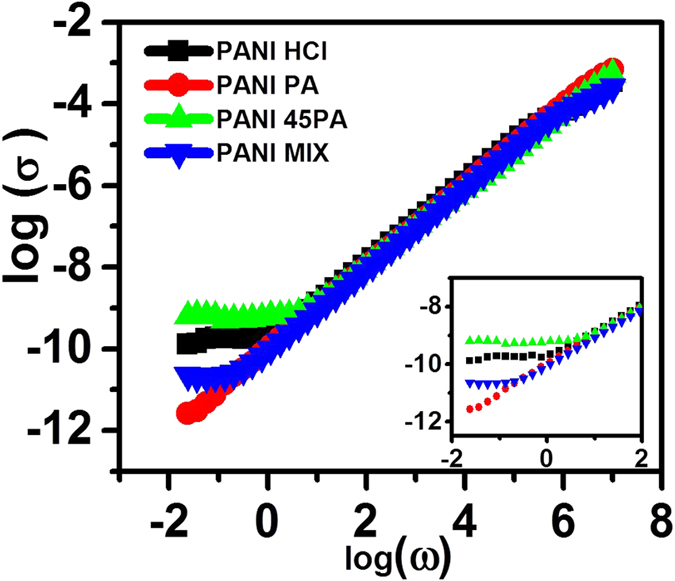
frequency dependent conductivity data for the four samples of interest inset: Lower frequency.

Figure 7 depicts the frequency dependant conductivity plots for the three cases of interest, along with the case of PANI MIX. All the four samples basically reflect similar general trend, namely a frequency independent conductivity over the low frequency regime and frequency dependant variation over the higher frequency range^54^,^55^. Significant differences are noted in the conductivity values at very low frequency *i.e.* near DC conductivity; the conductivity value being the highest for the PANI 45PA sample, which is consistent with the DC four probe measurement data mentioned earlier. The frequency at which the low and high frequency behaviours depart is called as the critical frequency *ω*_*c*_^56^. Higher *ω*_*c*_ reflects a more metallic behaviour^57^,^58^,^59^. It is observed that in the case of PANI MIX, *ω*_*c*_ value lies in between the values for PANI HCl and PANI PA, whereas in the PANI 45PA case it has the maximum value. Thus, the electrical behaviour of PANI 45PA is characteristically different from that of PANI MIX, the two materials differing at the molecular level.

Although PANI HCl must, in principle, have a higher conductivity than all other cases, this can be true only within the uncoiled polymeric strands. However, as a bulk electrode or a film the intra- and inter-fiber transport can be impeded by morphological factors, rendering an effectively lower conductivity than PANI 45PA. The PA in PANI 45PA is involved in cross-linking the binary doped polymer segments that do not allow polymer chains to coil along their propagation length to great extent and can allow the chains to exist in uncoiled conformation *w.r.t.* each other over long distances, which would not be the case in PANI HCl. Hence this concomitant effect leads to improvement in the effective conduction in the system. Thus, the 3D interconnected chain structure in PANI 45PA holds a robust morphology which allows multiple routes for charge carriers to flow hassle-free.

It is also remarkable that accessibility (morphology and pore size distribution) factor plays a major role in improvising the capacitance of PANI 45PA which might has almost half the number of electroactive centres as compared to PANI HCl. Thus, our synthetic strategy enables concurrent tuning of electrical conductivity (PANI HCl) and morphology (PANI PA) rendering strong improvements in all aspects of charge storage, especially at higher current rate which was not observed previously.

Another important point to ruminate is the polymerisation mechanism in the case of binary doping. It is proposed that polymerisation mechanism in the case of binary doping is quite similar to previous protonic acid doping mechanism^60^. Initially radicals corresponding to respective anions are formed which then couple randomly and subsequently resulting in the polymer. Referring to Fig. S6 A, Step 1 is the oxidation of monomers. Radical cations are formed as a result of oxidation in the acidic medium. Such radicals exist in two resonating forms. Step 2 (Fig. S6B) involves radical coupling and re-aromatisation. The radicals and their resonating structures formed in the above step react with each other leading to coupled product which then is reduced to form reactive radicals. Coupling is head to tail. Step 3 (Fig. S6C) includes chain propagation and formation of oligomers from dimers and monomers of different anions. It is proposed that during this step, radicals with different associated anions undergo coupling. In principle this hetero-anion-radical coupling can occur during the second step. It is also possible that PA anions can be associated with two different aniline radicals which is the origin of cross-linking. At this stage the steric nature of dopant and whole chain will determine the final morphology. This step further leads to the formation of pernigraniline. Reduction of pernigraniline by unreacted aniline molecules occures in step 4 (Fig S6D). Since pernigraniline is the state with highest oxidation level, it is highly reactive. To stabilize this state, unreacted aniline molecule reduce this to form the emeraldine state which is the green conducting phase. The order in which these anions appear on the chain is random and is difficult to assign but since chains are doped, it results in emeraldine state.

## Conclusion

Inorganic (HCl) and organic (phytic acid) co-doping renders a substantial and positive enhancement in the context of both the quality factors for the supercapacitor electrodes, namely high conductivity and porous morphology. An excellent performance is realized for the case of 55 HCl: 45PA co-doped sample with a capacitance of about 350 F g^−1^, which is found to be nearly constant over the wide range of current densities 1 A g^−1^ to 40 A g^−1^. This study highlights the significance of concurrent optimization of morphological (micro/meso porosity, surface area) and transport features of a superconducting electrode material in rendering the best performance and stability to the said device.

## Methods

### Synthesis of binary doped polyanilines

In order to understand the simultaneous effect of both dopants, polyaniline samples with various compositions of PA: HCl namely 0, 20, 45, 60, 80 and 100% PA were synthesized. Here xPA stands for x% of aniline neutralised by PA in the beginning. Aniline was distilled prior to use and rest of the chemicals were used as received. Aniline to APS mol ratio was 4:1 in all the cases. Typically in PANI 20PA case, in the first vial ‘**A’** 3 mL water+0.2 mL aniline were mixed and PA was added to get a clear solution. In another vial ‘**B’** 3 mL water + 0.8 mL aniline were mixed, sonicated and HCl was added till a clear solution was obtained. This step makes sure that 20% aniline is fully protonated by PA and 80% aniline is protonated by HCl. In a third vial ‘**C’** APS was dissolved in 3 mL water. All these 3 solutions were cooled to 5 °C. After an hour solutions in **A** and **B** vials were mixed and stirred for 10 s. Immediately, vial **C** solution was added to the above mixture and stirred for another 10 s and then kept unstirred at 5 °C. After an hour polyaniline was washed with water and methanol, and then dried. The same protocol was followed for rest of the different cases of the PA: HCl compositions. The yields in all cases were in the range 75–78%.

### Electrodes fabrication

Toray carbon paper (0.3 mm) was used for making electrodes and only ethanol was used as solvent. The ratio of the active material to SuperP to binder was 85:15:5. In all the cases, first the binder was dissolved in 1 mL ethanol, then SuperP was added, and this mixture was sonicated for 10 min at ~10 °C. Then the active material was mixed and further sonicated for half an hour at the same temperature. This step ensures proper and uniform mixing of all the components. To make paste of the required texture, it was ground for 15–20 min and then drop-casted on Toray paper having an area of 1 cm^2^ followed by drying at 80 °C for 12 h. The loading was in the range of 1 mg–1.5 mg.

### Electrochemical measurements

Electrochemical measurements were carried on Autolab PGSTAT 30 (ECO Chemie) with least count of 0.1 mA. Electrolyte used was 1 M H_2_SO_4_. A 3 electrode system was specifically used only for cyclic voltammetry and impedance spectroscopy measurements to understand the detailed electrochemical behaviour of the samples. Ag/AgCl electrode was used as reference and Pt as counter. Electrochemical Impedance Spectroscopy (EIS) was carried out in the frequency range 1 mHz to 10^4^ Hz. For charge discharge studies symmetrical two electrode system was preferred. The voltage window was from 0 to 0.7 V.

### Tapping Density measurements

The tapping density was calculated based on the volume occupied by a sample for a weight (m) after tapping, which is the same for the rest of the samples. A plastic measuring cylinder of capacity 10 mL with diameter of 1 cm was filled with 1 g of PANI HCl and tapped from constant height of 5 cm; tapping was repeated until it showed a constant volume (V). Tapping was free fall. The tapping density was calculated by the formula T_d_ = (m)/(V). Similar procedure was followed for rest samples.

### Electrical measurements

All the four selected samples of constant weight of 300 mg were pressed under constant pressure of 10 Tonns using hydraulic system for a minute. Before using, these pellets were dried in vacuum at 60 °C for 24 h. Electrical conductivity was measured on Novacontrol Microtonic Alpha machine at room temperature.

## Additional Information

**How to cite this article**: Gawli, Y. *et al.* 3D Polyaniline Architecture by Concurrent Inorganic and Organic Acid Doping for Superior and Robust High Rate Supercapacitor Performance. *Sci. Rep.* **6**, 21002; doi: 10.1038/srep21002 (2016).

## Supplementary Material

Supplementary Information

## Figures and Tables

**Figure 1:**
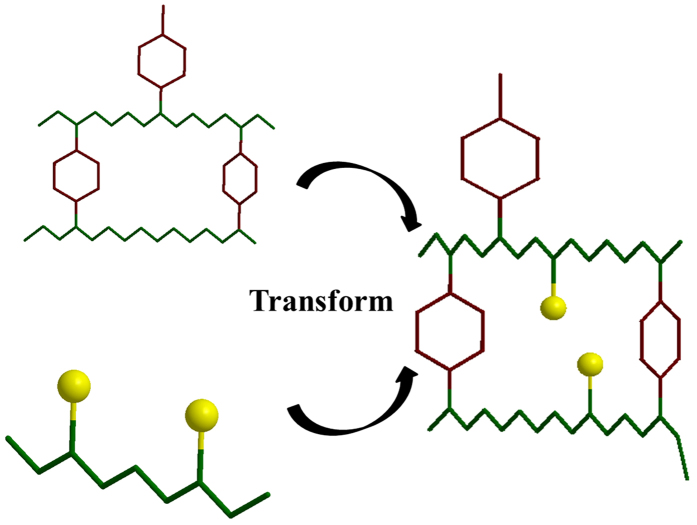
3D PANI Architecture by binary (weak and strong) acid doping (Yellow: Chlorine, Red: phytic acid, green: polymer chain).

**Table 1:** Electrochemical Impedence Spectroscopy (EIS) parameters of four samples.

Material	ESR (Ω)	R_ct_ (mΩ)	Knee Frequency (Hz)
PANI HCl	1.08	460	7.0
PANI PA	1.10	870	4.0
PANI 45PA	0.92	420	16.0
PANI MIX	1.32	1100	2.9
