# Supplementary material for: 3D Polyaniline Architecture by Concurrent Inorganic and Organic Acid Doping for Superior and Robust High Rate Supercapacitor Performance
Source: Sci Rep. 2016 Feb 12;6:21002. doi: 10.1038/srep21002 (PMC4751491; doi:10.1038/srep21002)
Supplement: Supplementary Information [file srep21002-s1.doc]

**3D Polyaniline Architecture by Concurrent Inorganic and Organic Acid Doping for Superior and Robust High Rate Supercapacitor Performance**

Yogesh Gawli,* ^a,b^ Abhik Banerjee,^a^ Dipti Dhakras,^a,b^ Meenal Deo,^a^ Dinesh Bulani,^c^ Prakash Wadgaonkar, ^a,b^ Manjusha Shelke,^a,b^ and Satishchandra Ogale * ^a, c^

^a^Physical and Materials Chemistry Division, National Chemical Laboratory (CSIR-NCL), Pashan, Pune, 411008, India

^b^Academy of Scientific and Industrial Research, (AcSIR) Anusandhan Bhavan, 2 , Rafi Marg, New Delhi, India

^c^Department of Physics and Centre for Energy Science, Indian Institute of Science Education and Research, Dr. Homi Bhabha Road, Pune 411008, India

Corresponding authors: satishogale@iiserpune.ac.in, [yogeshgawli5@gmail.com](mailto:yogeshgawli5@gmail.com)


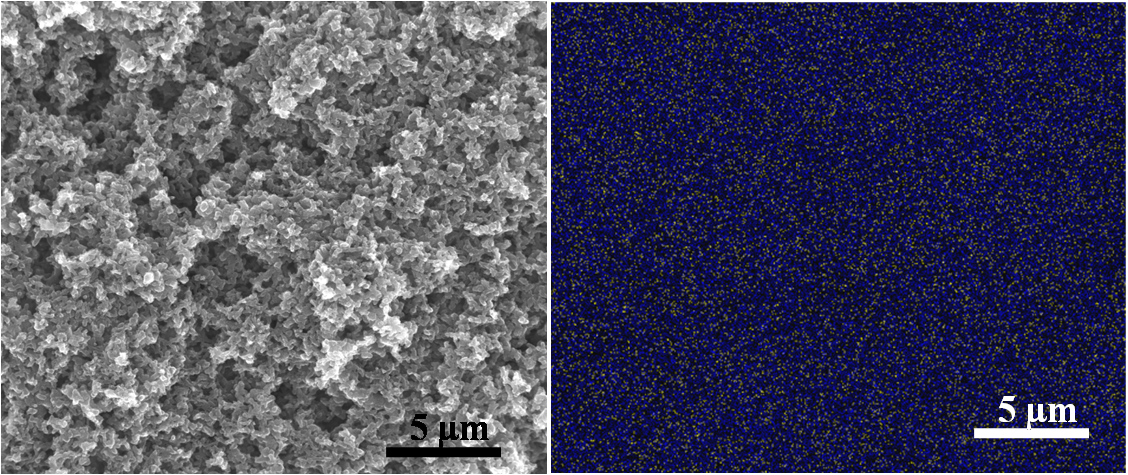


**Figure S1.** SEM elemental map of PANI 45PA suggesting homogeneous dispersion of

phosphorus (blue dots) and chlorine (yellow dots).

**Figure S2**. FTIR of PANI PA and PANI 45PA . This confirms emeraldine salt state of PANI 45PA

**Figure S3.** Tapping density of all samples as a function of % PA.

**Figure S4.**  Discharge time curves of PANI HCl(a), PANI PA(b), PANI 45PA(c), PANI MIX (d)


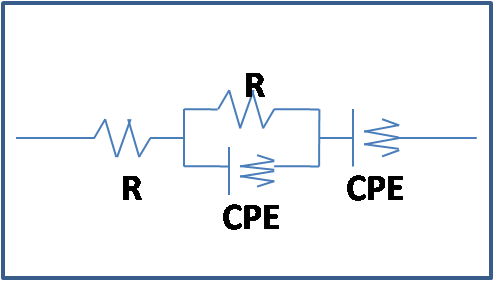


**Figure S5.**  Fitted circuit for EIS measurements

**Table S1.**  Comparison of this study with previous reports

| Ref # | *(Synthesis method)* | Specific Cap at 1A/g | Specific Cap at 10A/g | At 40A/g | % Retention  From 1A/g to 10A/g |
| --- | --- | --- | --- | --- | --- |
| 1 | Polyaniline nanofibers | 200 | ~10 | NA | 5 |
| 2 | Polyaniline nanoparticle | ~625 | ~470 | NA | 75 |
| 3 | Electrospun template and polyaniline fibers | 600 | ~350 | NA | 41 |
| 4 | Polyaniline on graphite | 2136 | ~1000 | NA | 24 |
| 5 | Polyaniline nanofibers | 428 | ~320 (9A) | NA | 70 |
| 6 | Polyaniline graphene | 800 | 600 | NA | 75 |
| 7 | Polyaniline hollow spheres | 400 | 200(5A/g) | NA | 50 |
| 8 | Polyaniline graphene composite | 600 | 450 | NA | 75 |
| 9 | Polyaniline film | 500 | 300 | NA | 60 |
| **10** | **This study (Solution process)** | **371** | **360** | **360** | **97** |

Additional TEM images of PANI 45PA


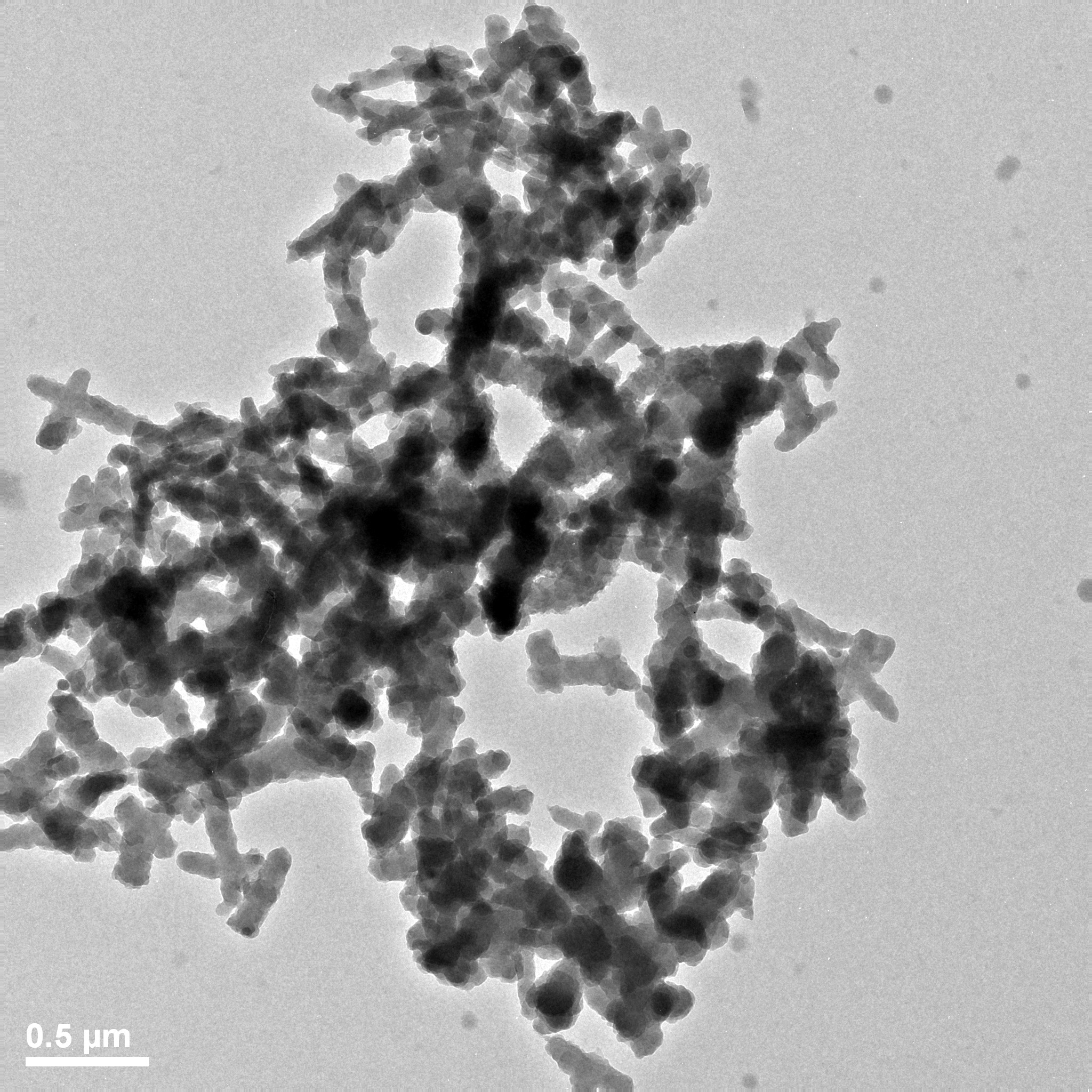

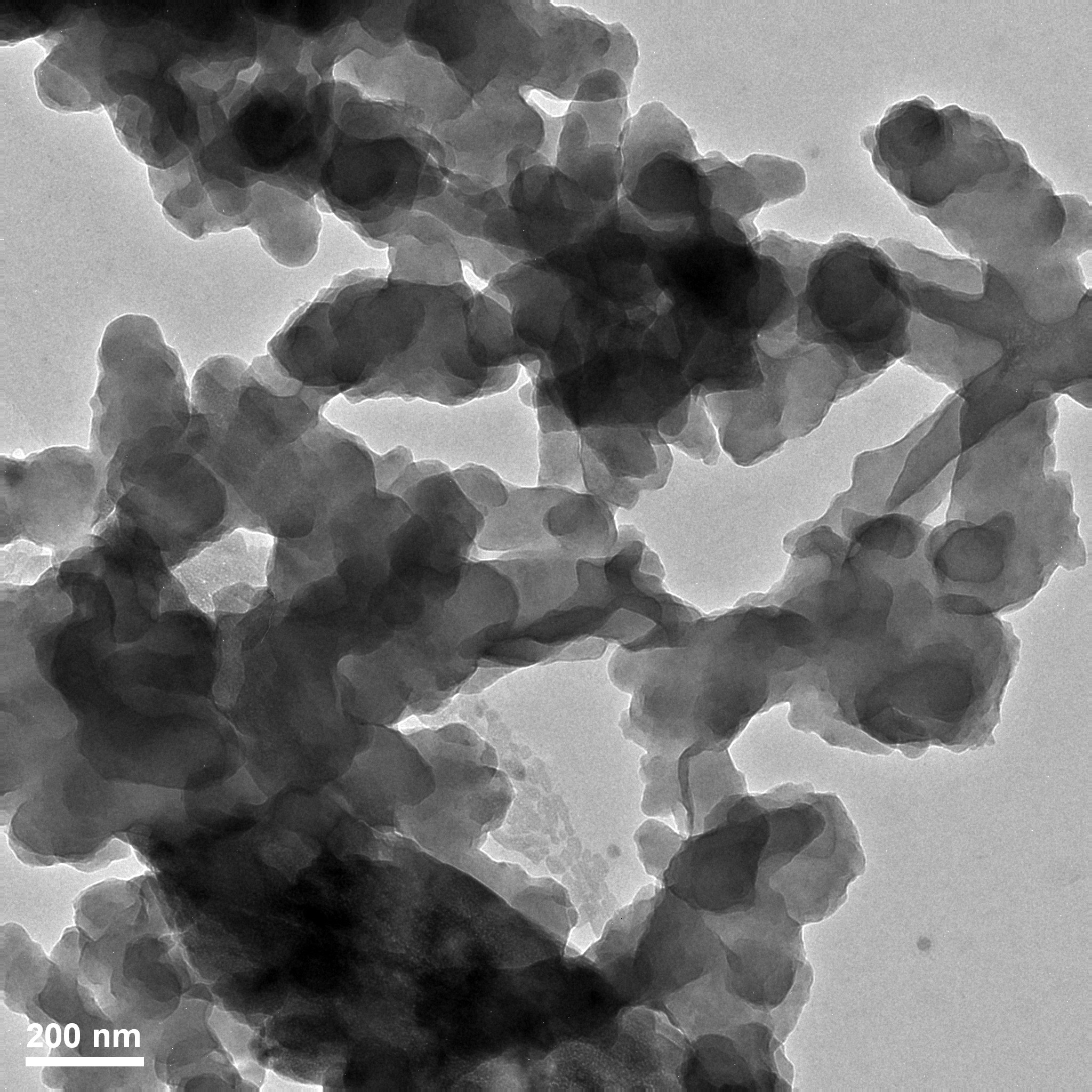


Fig S5: TEM images of PANI 45PA (left) at 500nm and (right) 200nm

Polymerisation mechanism Fig S6

(A) Oxidation of monomers (X^-^ = Cl^-^  or PA^n-^)

(A) Oxidation of monomers

Fig S6 (B) Radical Coupling and re-aromatisation

Start of crosslinking

Fig S6 (C) Chain propogation

Fig S6 (D) Reduction of pernigraniline by unreacted aniline molecule.

**References**

1. Park, H., *et al*, Anisotropic Growth Control of Polyaniline Nanostructures and Their Morphology-Dependent Electrochemical Characteristics *ACS Nano,* **6***,* 7624-7633 (2012).

2. Anothumakkool, B., *et al*. Design of a High Performance Thin All-Solid-State Supercapacitor Mimicking the Active Interface of Its Liquid-State Counterpart *ACS Appl.Mater. Inter.,* **5***,* 13397-13404, (2013).

3. Miao, Y., Fan, W., Chen, D., Liu T., High-Performance Supercapacitors Based on Hollow Polyaniline Nanofibers by Electrospinning *ACS Appl. Mater. Inter.,* **5***,* 4423-4428 (2013).

4. Li X., *et al*. Microwave-Assisted Chemical-Vapor-Induced in Situ Polymerization of Polyaniline Nanofibers on Graphite Electrode for High- Performance Supercapacitor *ACS Appl. Mater. Inter.,* **6,** 19978-19989 (2014).

5. Mi, H., Zhang, X., Yang, S., Ye, X., Luo, J. Polyaniline nanofibers as the electrode material for supercapacitors *Mater. Chemi. Phy.,* **112,** 127-131 (2008).

6. He S. *et al*, Needle-like polyaniline nanowires on graphite nanofibers: hierarchical micro/ nano-architecture for high performance supercapacitors *J. Mater. Chem . A,* **22,** 5114-5120 (2012).

7. Ning, G., *et al*. Three-dimensional hybrid materials of fish scale-like polyaniline nanosheet arrays on graphene oxide and carbon nanotube for high-performance ultracapacitors *Carbon,* **54**, 241-248 (2013).

8. Fan, W., Graphene-Wrapped Polyaniline Hollow Spheres As Novel Hybrid Electrode Materials for Supercapacitor Applications *ACS Appl. Mater. Inter.,* **5,** 3382-3391 (2013).

9. Cong, H., Ren, X., Wang, P., Yu, S. Flexible graphene–polyaniline composite paper for

high-performance supercapacitor *Energy Environ. Sci.,* **6,** 1185-1191 (2013).
